# A Deep Learning Radiomics Analysis for Survival Prediction in Esophageal Cancer

**DOI:** 10.1155/2022/4034404

**Published:** 2022-03-24

**Authors:** Junxiu Wang, Jianchao Zeng, Hongwei Li, Xiaoqing Yu

**Affiliations:** ^1^Data Science and Technology, North University of China, Taiyuan, Shanxi 030051, China; ^2^Department of Computer Engineering, Taiyuan Institute of Technology, Taiyuan, Shanxi 030008, China; ^3^Shanxi Tumor Hospital, Taiyuan 030013, Shanxi Province, China

## Abstract

The purpose of this study was to explore the deep learning radiomics (DLR) nomogram to predict the overall 3-year survival after chemoradiotherapy in patients with esophageal cancer. The 154 patients' data were used in this study, which was randomly split into training (116) and validation (38) data. Deep learning and handcrafted features were obtained via the preprocessing diagnostic computed tomography images. The selected features were used to construct radiomics signatures through the least absolute shrinkage and selection operator (LASSO) regression, maximizing relevance while minimizing redundancy. The DLR signature, handcrafted features' radiomics (HCR) signature, and clinical factors were incorporated to develop a DLR nomogram. The DLR nomogram was evaluated in terms of discrimination and calibration with comparison to the HCR signature-based radiomics model. The experimental results showed the outperforming discrimination ability of the proposed DLR over the HCR model in terms of Harrel's concordance index, 0.76 and 0.784, for training and validation sets, respectively. Also, the proposed DLR nomogram calibrates and classifies better than the HCR model in terms of AUC, 0.984 (vs. 0.797) and 0.942 (vs. 0.665) for training and validation sets, respectively. Furthermore, the nomogram-predicted Kaplan–Meier survival (KMS) curves differed significantly from the nonsurvival groups in the log-rank test (*p* value <0.05). The proposed DLR model based on conventional CT images showed the outperforming performance over the HCR signature model in noninvasively individualized prediction of the 3-year survival rate in esophageal cancer patients. The proposed model can potentially provide prognostic information that guides and helps the clinical decisions between observation and treatment.

## 1. Introduction

Esophageal cancer (EC) is the eighth most common malignancy and the sixth most common disease-related cause of death worldwide [1, 2]. The incidence of esophageal cancer is notably high in Asia and Iceland, as well as the United Kingdom and the United States [3–5]. In order to systemically control the disease, radiotherapy and neoadjuvant chemotherapy are commonly combined with surgery [6–8]. Regardless of its benefit for severe patients with a low survival rate, an aggressive treatment plan, including multiple cycles of treatment and adjuvant chemotherapy, is not suitable for the other patients with esophageal cancer [9]. Pre-identification for such patients having a low survival rate before surgery can help provide other suitable treatment regimens for these patients [10]. Therefore, identification of patients having a lower survival rate is vital to take benefit from additional treatment.

Radiomics features [11, 12] have been widely used as an extremely useful tool in quantitative analysis of medical imaging and in medical diagnosis [13, 14]. The traditional radiomics utilized handcrafted features, such as tumor shape and texture, obtained from medical images [11]. However, such handcrafted low-order features are not suitable to define intrinsic characteristics of intratumor imaging heterogeneity, limiting the applicability of the radiomics model [11–13]. Furthermore, the construction of handcrafted features is limited within the known knowledge of medical imaging.

Deep learning, especially CNN, has recently achieved promising results in medical image analysis [15–18]. Deep networks consist of multiple layers that can be learned from data [19, 20]. For example, prognostic tumor features can be extracted through hierarchical convolution operation on the medical images [21–24]. Compared with handcrafted features, DLR features contain more important tumor information that may help diagnose [25–27].

However, unlike handcrafted radiomics studied widely for radiological diagnosis and prediction [28], the application of deep learning in predicting overall survival in esophageal cancer has not been thoroughly explored yet. Hence, this study aims to develop and validate the deep survival prediction model based on a radiomics nomogram for individualized prediction of three years' overall survival in patients with esophageal cancer.

It is worthwhile to highlight three aspects of the contributions here. (1) This study investigated DLR features in the survival prediction of patients with esophageal cancer. Unlike the traditional handcrafted features, clinical target-oriented DLR features can be automatically learned from data. (2) Both HCR features and DLR features are considered in the prediction model to characterize the esophageal lesions thoroughly. The main reason is that the DLR and HCR can describe imaging heterogeneity of esophageal lesions in different levels. In particular, HCR comprise shape features, first-order statistics, and texture features; DLR contains the “real-world” textures, which are extracted from the pretrained DenseNet-169 network via transfer learning strategy. (3) This study develops a noninvasive predictive model that combines deep learning-based radiomics features, handcrafted features, and clinical factors to predict survival rates within three years at diagnosis of esophageal cancer. The DLR nomogram survival prediction of esophageal cancer patients can allow more proper treatment. The experimental results showed that the DLR nomogram outperforms the HCR model and the clinical model.

## 2. Materials and Methods

### 2.1. Patients

Esophageal cancer patients at Shanxi Cancer Hospital were the subject of our retrospective study. The patients were included according to the inclusion criteria: (a) patients who had pathologically confirmed esophageal cancer, (b) a standard CT scan performed before any treatment, and (c) clinical characteristics available. The patients were excluded with the following criteria: (a) too poor CT image quality, which may affect the diagnosis of the patient, (b) patients who had chemotherapy treatment at another institution, and (c) patients who are also suffering from other cancers.

The survival group includes patients who survived more than three years since the treatment, whereas the nonsurvival group includes patients who died within three years. A total of 154 esophageal cancer patients diagnosed from November 2012 to February 2015 participated in our retrospective study. Those data were grouped into two sets: training (116) and validation (38) data at a ratio of 3 : 1.

Baseline clinical data were collected via the electronic medical record system (EMRS) [29], including gender, BMI, age, M-stage, N-stage, T-stage, overall stage, and planning target volume (PTV). The picture archiving and communication system (PACS) was used to obtain CT images. The dataset was constructed and evaluated in April 2019, and all enrolled patients were followed for at least 3 years. The Institutional Review Board approved the study.

### 2.2. CT Image and Region of Interest (ROI) Acquisition

General Electric Light Speed RT16 was used for scanning, with a CT thickness of 5 mm. The primary tumor volumes for radiotherapy planning were set as the ROI to quantitatively analyze the images. Two skilled radiologists manually selected the three-dimensional tumor ROI using the software package 3D Slicer [30].

Training CT images were preprocessed to avoid accuracy degradation of DL models caused by noises introduced with the interval change, which include resampling, rescaling, and voxel normalization. Those CT images were reconstructed with a matrix of 512  × 512 and 0.5 × 0.5 mm^2^ pixel size, and the resampling with cubic interpolation to 1 × 1 × 1 mm^3^ pixels was conducted, minimizing CT images variabilities [31].

The tumor area was located with a rectangle bounding box that covers the primary tumor area. The ROI for each patient was obtained with three cropped consecutive slices to avoid the bias of manual segmentation that affects the location of a bounding box. Lastly, the tumor image was resized to 224 × 224 × 3 voxels.

### 2.3. Radiomics Feature Extraction

Phenotypic differences between tumors can be captured by a large number of quantitative radiomics features. In this study, deep learning features and handcrafted features were extracted to quantify tumor phenotype to enhance the learning efficiency of the radiomics model. Those two feature sets have complementary advantages that can be combined to improve the model. Also, expert knowledge on the esophageal cancer lesion can be reflected with shape and texture features. On the contrary, the high-level DLR features can significantly represent complex spatial features in both global and local perspectives.

The handcrafted feature extraction algorithm was standardized by referring to the Image Biomarker Standardization Initiative (IBSI) [32–34] and Radiomics Ontology [35]. For each CT ROI, 1,670 handcrafted features were extracted using Python implementation, including 18 first-order statistics, 16 geometric, and 1,564 texture features. The textural features include 14 gray-level dependence [36], 23 gray-level co-occurrence [37], 16 gray-level run-length [38], 16 gray-level size-zone [39], and 5 neighborhood gray-tone difference [40] matrices. Refer to the supplementary appendix of Lambing [41], for mathematical definitions of those features.

**Figure 1:**
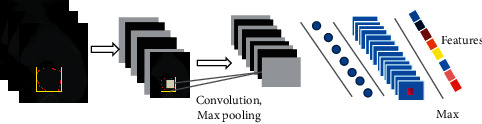
DLR feature extraction process.

The DenseNet-169, designed for the image classification task, was adopted to extract DLR features. In the training cohort, data augmentation approaches including random rotation, random shear, and random zoom were employed before the training procedure. The deep learning model was pretrained on the ImageNet dataset, one of the largest image datasets, and then fine-tuned in a transfer learning strategy to avoid the overfitting problem [42]. The network was trained with cross-entropy loss function and Adam optimizer with a learning rate of 0.0001, a batch size of 16, and a regularization weight of 0.0001. The network was implemented on Keras (https://keras.io/) with the TensorFlow library as the backend (https://www.tensorflow.org/). As depicted in Figure 1, the tumor ROI was fed into the DenseNet-169, and the outputs of hidden layers were collected to obtain 1,664 features in total.

### 2.4. DLR Signature Building

In order to obtain the most effective feature, three stages of feature selection were carried out. First, features (*p* < 0.05) were obtained through the Mann–Whitney U (MWU) test. Then, the features were sorted based on the mutual information (MI) between features and the survival status using the minimum redundancy maximum correlation (mRMR) scheme [43]. It should be noted that, in this study, only the top 50 features in mRMR were retained. Lastly, the dimension of features is reduced by the LASSO to obtain optimal features [44]. The survival-related features were retained while the other features were removed by LASSO regression. The 10-fold cross validation was conducted with 100 iterations in LASSO regression. The obtained features are used to construct the DLR signature, and the HCR signature was constructed in a similar way for comparison.

### 2.5. DLR Nomogram Construction

A DLR nomogram was built by integrating DLR signature, HCR signature, and clinical features with a multivariable logistic regression model. Backward stepping selection was used with information criterion of Akaike as the stopping rule [45]. The variable multicollinearity in the multiple logistic regression model was checked by the variance inflation factor (VIF), where VIF > 10 indicates high multicollinearity [46]. A DLR nomogram was then built based on the multivariate logistic analysis, predicting the individual probability of survival in the training dataset.

### 2.6. Evaluation of the DLR Nomogram

Harrel's C -index was employed to evaluate the discrimination ability of the DLR nomogram in both training and testing datasets. The bootstrap method was used to resampling 1,000 times, and the C index in both cohorts was calculated with 95% confidence intervals. The AUC, accuracy, specificity, and sensitivity were calculated on the plotted ROC curves. The calibration ability of the DLR nomogram was evaluated using the calibration curve that depicts the consistency between predicted and actual survival probabilities. Hosmer–Lemeshow (HL) test [47] and decision curve analysis (DCA) [48] were utilized to evaluate the fitting accuracy and robustness of the DLR nomogram, respectively. Furthermore, KMS curves were constructed to predict survival status. Accordingly, the patients were predicted as survival or nonsurvival, and then, the difference in survival curves between the two groups was evaluated using the log-rank test.

### 2.7. Statistical Analysis

All the statistical analyses were conducted with R software (version 4.0.3; http://www.Rproject.org). MWU and Chi-square tests were adopted for univariate analysis, and Spearman's correlation rank was employed for correlation. The penalty parameter (*λ*) was tuned by LASSO logistic regression model. This study used the following packages for each analysis. “glomnet” package: LASSO logistic regression, “rms” package: nomograms and calibration plots, “ResourceSelection” package: HL test, “car” package: VIFs calculation, “survivalROC” package: AUC analysis, “survminer” package: KMS analysis, and “dca.R” function: DCA performance. This study utilized a bilateral statistical significance level *p* value <0.05.

## 3. Results

**Figure 2:**
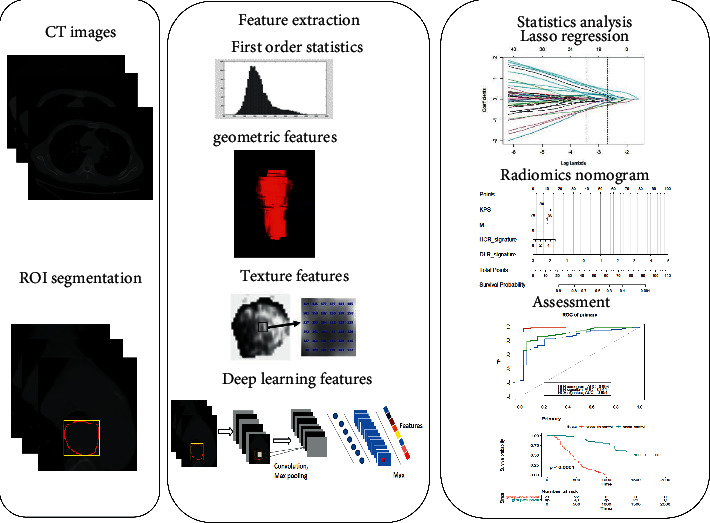
Schematic diagram of this study.

Figure 2 depicts the schematic diagram of the study.

### 3.1. Clinical Characteristics

Table 1 summarizes the clinical characteristics of the training and validation cohorts, where the Chi-square test (*p* value is 0.572) for two data shows no significant observable difference in the survival rate, 30.2% and 36.8%, for training and validation, respectively.

### 3.2. DLR Signature

Fifty features were obtained for each patient after survival-unrelated and redundant feature removal from 1,664 DLR features. Then, based on the training cohort, 33 potential predictors were selected by LASSO regression. Parameter (*λ*) selection and coefficients of LASSO are given in Supplementary Material (II). The DLR signature was constructed using the selected features.

The results show that scores of the survival group were higher than the nonsurvival group with a significant difference in terms of DLR signatures (1.10 ± 0.75 vs. −2.22 ± 0.75) in the training cohort and (0.15 ± 1.11 vs. −1.90 ± 1.12) in the validation cohort. MWU test was used with a *p* value <.001. Also, a significant correlation between DLR signature and survival status was found (C index: 0.729, *p*=0.035 in the training data, and C index: 0.766, all *p* < 0.001 in the validation data).

The LASSO algorithm selected 18 handcrafted features to build HCR signatures. HCR signatures were also significantly different between survival and nonsurvival groups. In the training data, 0.68 ± 1.12 vs. −0.50 ± 1.03, *p* value <.001, MWU test, and in the validation data, 0.80 ± 1.26 vs. −0.23 ± 1.48, *p* value = 0.035, MWU test. HCR feature selection by LASSO regression is described in detail in Supplementary Material (I).

### 3.3. DLR Nomogram

**Figure 3:**
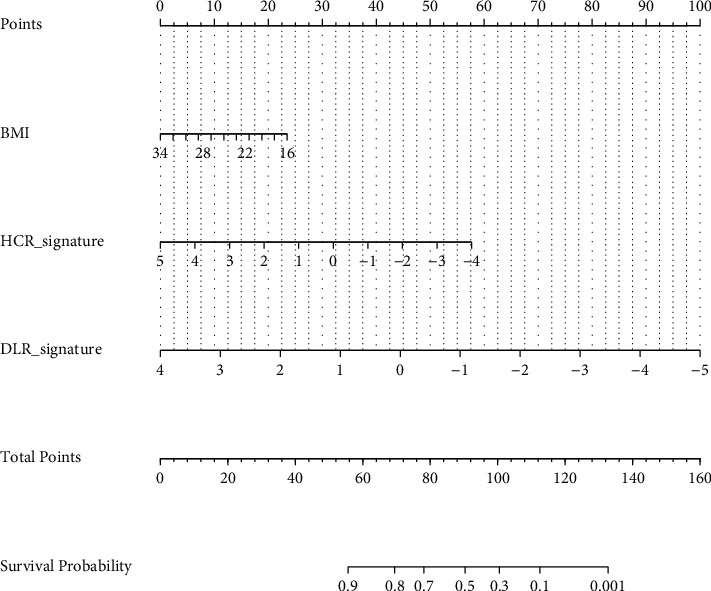
The construction of the DLR nomogram.

The DLR signature, HCR signature, and BMI were combined to construct a DLR nomogram, as shown in Figure 3. The VIFs of DLR signature, HCR signature, and BMI were 1.45, 1.41, and 1.07, respectively, indicating no severe collinearity in the regression model.

**Figure 4:**
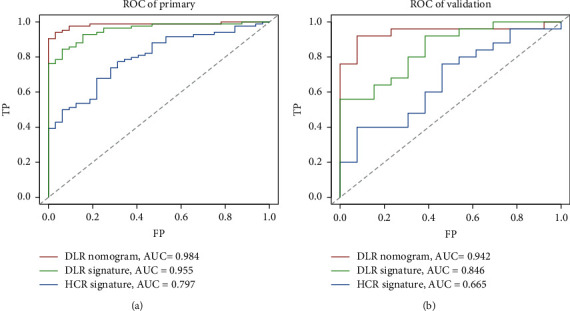
ROC curves for the DLR and HCR signatures for (a) training data and (b) validation data.

Figure 4 depicts ROC curves of the DLR nomogram for the DLR signature model and HCR signature model. The AUC was 0.984, 0.955, and 0.797 for the DLR nomogram, DLR signature model, and HCR signatures' model, respectively, in the training data. In the validation data, the AUC was 0.942, 0.846, and 0.665 for the DLR nomogram, DLR signature model, and HCR signatures' model, respectively. The results indicate that the DLR nomogram model provides better discrimination ability (Harrel's concordance index, 0.76 and 0.784, for the training and validation data, respectively).

**Figure 5:**
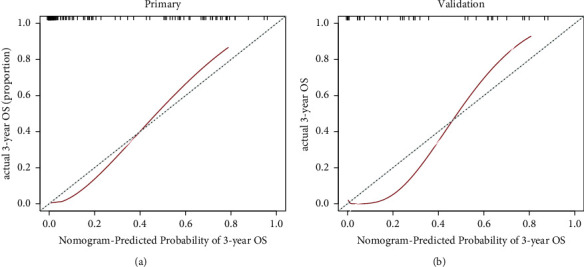
Calibration curves for the DLR nomogram for (a) training data and (b) validation data, where the *x*-axis and *y*-axis represent the predicted and actual rates. The solid red line is the performance of the DLR nomogram, and the dashed blue line is the ideal prediction. Closer the solid red line is to the dashed blue line, more accurate the prediction of the model.

Figure 5 depicts the calibration curves, showing the consistency between predicted and actual survival rates. A nonsignificant statistic of the training cohort (*p* value = .563, HL test) showed no deviation from the ideal ﬁt. In the validation cohort, the 3-year survival rate was also well-calibrated (*p* value = .648, HL test).

**Figure 6:**
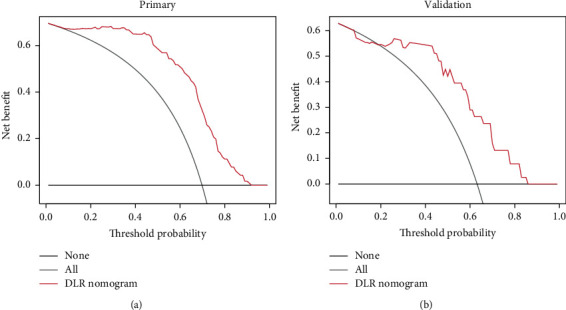
DCA for the DLR nomogram in (a) training and (b) validation data. The black and gray lines represent the hypothesis that all patients die and that no patient dies within three years, respectively. The red line represents the net benefit of the DLR nomogram.

**Figure 7:**
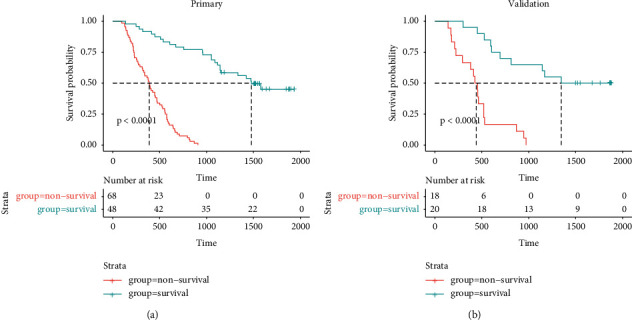
KMS curves of the predicted survival and nonsurvival groups in (a) training and (b) validation data.

The DCA examined the clinical outcomes based on threshold probability at which a net benefit could be derived.  Figure 6 depicts the DCA of the DLR nomogram, showing that the DLR nomogram obtained outstanding net benefits over the other strategies: treat-all-patients and treat-none strategies. A significant difference (*p* value <.05, log-rank test) between prediction survival and nonsurvival groups was found in KMS curves (Figure 7).

## 4. Discussion

Treatment planning can be further individualized via preoperative prediction of three-year survival. In previous studies, handcrafted features were analyzed to predict survival rates. However, due to the limited feature extraction ability, the prediction accuracy was not high enough. In order to overcome such a limitation, this study investigated DLR features in the survival prediction of patients with esophageal cancer. Unlike the traditional handcrafted features, clinical target-oriented DLR features can be automatically learned from data [49].

Intratumor heterogeneity has been considered a potential prognosis factor. The DRL feature extraction can robustly characterize the intratumor heterogeneity noninvasively from the medical images [26]. The experimental results showed that the use of DLR features contributed to the performance of the model, which is also supported by recent studies that high-dimensional features can preserve more detailed cancer information, making them more sensitive when assessing survival status [24]. Therefore, by combining these DLR features, a DLR nomogram survival prediction of esophageal cancer patients can allow more proper treatment. The experimental results showed that the DLR nomogram outperforms the HCR model and the clinical model.

This study has several limitations, described as follows. First, only 154 patients were available for a three-year follow-up analysis. A larger amount of data is required to improve the performance of the model. Second, all the patients were collected to form a single-center, thereby limiting the generalizability of the DLR model. A more diverse dataset is required to validate the robustness and reproducibility of the DLR model. Third, our study did not consider genetic markers. Multiple factors should be considered for more personalized treatment, including biology, pathology, genomics [24, 26, 42–52], and imaging biomarkers [53]. In addition, this study was limited to CT images despite the essentiality of MIR images in surgical planning due to their excellent resolution for soft tissues. The focus should, therefore, be given towards developing an additional model combining CT and MRI image features. Finally, the primary tumor volumes were manually delineated for feature extraction. Even though the delineations are commonly used with confirmation by another radiation oncologist in radiotherapy planning, previous studies showed that semiautomatic tumor segmentation could reduce interobserver variability and therefore is more suitable for radiomics studies [54].

## 5. Conclusions

This study details the development of a noninvasive predictive model that combines deep learning-based radiomics features, handcrafted features, and clinical factors to predict survival rates within three years at diagnosis of esophageal cancer. The performance of the proposed DLR nomogram is superior to the traditional radiomics model in terms of Harrel's concordance index and AUC. The calibration curves show the good prediction performance of the nomogram. The nomogram-predicted Kaplan–Meier survival (KMS) curves differed significantly from the nonsurvival groups in the log-rank test (*p* value <0.05). The proposed model can present the basis for clinicians to make better treatment decisions and personalized diagnoses. Future works will include the model improvement based on larger data and complementary clinical factors.

## Figures and Tables

**Table 1:** Clinical characteristics of esophageal cancer patients.

	Training cohort (*n* = 116)			Validation cohort (*n* = 38)		
Characteristic	Nonsurvival (81)	Survival (35)	*p*	Nonsurvival (24)	Survival (14)	*p*
Gender						
Male: female	51 : 30	23 : 12	0.836	12 : 12	6 : 8	0.745
Age	67.96 ± 9.41	68.83 ± 8.80	0.644	66.54 ± 9.65	67.36 ± 7.09	0.785
BMI	21.70 ± 3.14	23.69 ± 4.25	0.006	23.04 ± 3.98	22.55 ± 3.24	0.702
T-stage						
T1	1 (1.2%)	2 (5.7%)	0.042	1 (4.2%)	0 (0.0%)	0.938
T2	20 (24.7%)	16 (45.7%)		9 (37.5%)	7 (50.0%)	
T3	43 (53.1%)	13 (37.1%)		10 (41.7%)	5 (35.7%)	
T4	17 (21.0%)	4 (11.4%)		4 (16.7%)	2 (14.3%)	
N						
N0	20 (25.0%)	14 (40.0%)	0.123	7 (29.2%)	6 (42.9%)	0.486
N1	60 (75.0%)	21 (60.0%)		17 (70.8%)	8 (57.1%)	
M						
M0	69 (85.2%)	35 (100.0%)	0.017	22 (91.7%)	14 (100.0%)	0.522
M1	12 (14.8%)	0 (0.0%)		2 (8.3%)	0 (0.0%)	
TNM						
I	0 (0.0%)	2 (5.7%)	0.002	0 (0.0%)	0 (0.0%)	0.766
II	27 (33.3%)	19 (54.3%)		10 (41.7%)	8 (57.1%)	
III	42 (51.9%)	14 (40.0%)		12 (50.0%)	5 (35.7%)	
IV	12 (14.8%)	0 (0.0%)		2 (8.3%)	1 (7.1%)	
PTV	379.62 ± 158.34	335.22 ± 179.33	0.186	382.56 ± 134.39	362.53 ± 166.27	0.687
HCR_signature	−0.50 ± 1.03	0.68 ± 1.12	<0.001	−0.23 ± 1.48	0.80 ± 1.26	0.035
DLR_signature	−2.22 ± 0.75	1.10 ± 0.75	<0.001	−1.90 ± 1.12	0.15 ± 1.11	<0.001

## Data Availability

The data used to support this research are included within the article.

## Supplementary Materials

Supplementary Materials (I) HCR feature selection using the least absolute shrinkage and selection operator (LASSO) logistic regression model. Figure S1. HCR feature selection using the LASSO logistic regression algorithm. (a) The penalization coefficient *λ* in the LASSO model was tuned by the binomial deviance minimization criteria. The binomial deviance metrics (the *y*-axis) were plotted against log(*λ*) (the bottom *x*-axis). The top *x*-axis indicates the number of predictors with the given log(*λ*). Red dots indicate average binomial deviance for each model at the given *λ*. Vertical bars through the red dots show the upper and lower values of the binomial deviance. The vertical black lines represent the optimal *λ*, where the model provides the best fit to the data. As a result, the optimal *λ* of 0.02373184 was selected. (b) LASSO coefficient profiles of the 50 radiomics features. For the optimal *λ*, eighteen features with nonzero coefficient were selected. (II) DLR feature selection using the least absolute shrinkage and selection operator (LASSO) logistic regression model. Figure S2. DLR feature selection using the LASSO logistic regression algorithm. (a) The penalization coefficient *λ* in the LASSO model was tuned by the binomial deviance minimization criteria. As a result, the optimal *λ* of 0.0445107 was selected. (b) LASSO coefficient profiles of the 50 radiomics features. For the optimal *λ*, thirty three features with nonzero coefficient were selected.
