# Supplementary material for: A Deep Learning Radiomics Analysis for Survival Prediction in Esophageal Cancer
Source: J Healthc Eng. 2022 Mar 24;2022:4034404. doi: 10.1155/2022/4034404 (PMC8970800; doi:10.1155/2022/4034404)
Supplement: Supplementary Materials — (I) HCR feature selection using the least absolute shrinkage and selection operator (LASSO) logistic regression model. Figure S1. HCR feature selection using the LASSO logistic regression algorithm. (a) The penalization coefficient λ in the LASSO model was tuned by the binomial deviance minimization criteria. The binomial deviance metrics (the y-axis) were plotted against log(λ) (the bottom x-axis). The top x-axis indicates the number of predictors with the given log(λ). Red dots indicate average binomial deviance for each model at the given λ. Vertical bars through the red dots show the upper and lower values of the binomial deviance. The vertical black lines represent the optimal λ, where the model provides the best fit to the data. As a result, the optimal λ of 0.02373184 was selected. (b) LASSO coefficient profiles of the 50 radiomics features. For the optimal λ, eighteen features with nonzero coefficient were selected. (II) DLR feature selection using the least absolute shrinkage and selection operator (LASSO) logistic regression model. Figure S2. DLR feature selection using the LASSO logistic regression algorithm. (a) The penalization coefficient λ in the LASSO model was tuned by the binomial deviance minimization criteria. As a result, the optimal λ of 0.0445107 was selected. (b) LASSO coefficient profiles of the 50 radiomics features. For the optimal λ, thirty three features with nonzero coefficient were selected. [file 4034404.f1.docx]

**Supplementary (if necessary)**

**І.HCR feature selection using the least absolute shrinkage and selection operator (LASSO) logistic regression model**


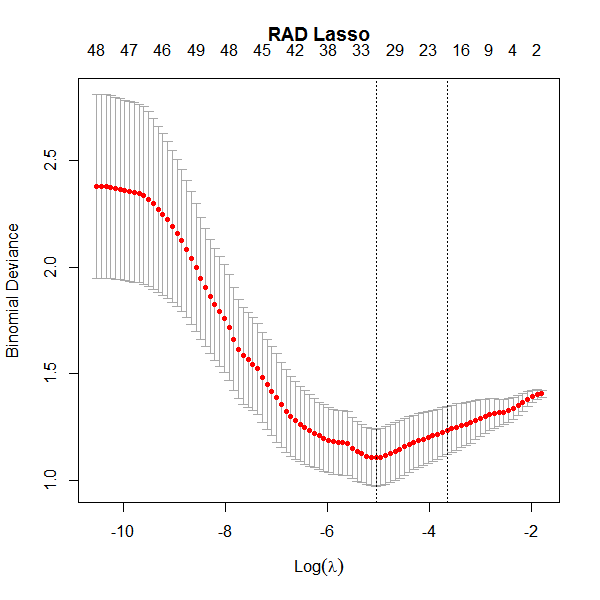

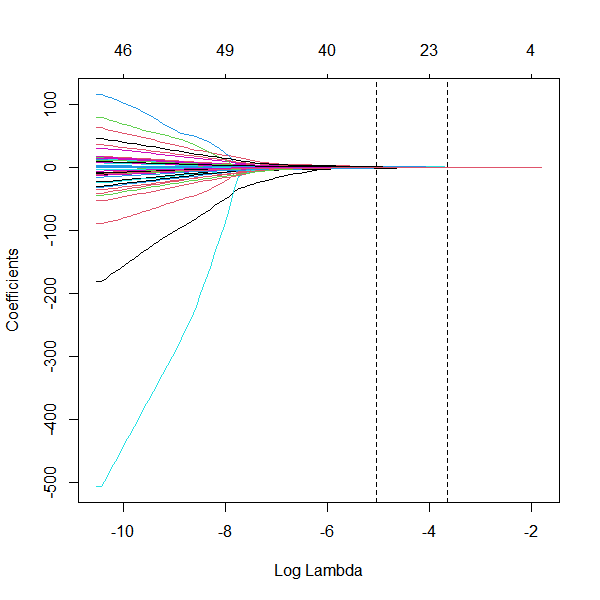


(a) (b)

**Figure S1** HCR features selection using the LASSO logistic regression algorithm. (a) The penalization coefficient λ in the LASSO model was tuned by the binomial deviance minimization criteria. The binomial deviance metrics (the y-axis) were plotted against log(λ) (the bottom x-axis). The top x-axis indicates the number of predictors with the given log(λ). Red dots indicates average binomial deviance for each model at the given λ. Vertical bars through the red dots shows the upper and lower values of the binomial deviance. The vertical black lines represents the optimal λ, where the model provides the best fit to the data. As a result, the optimal λ of 0.02373184, was selected. (b) LASSO coefficient profiles of the 50 radiomics features. For the optimal λ, eighteen features with nonzero coefficient were selected.

**П. DLR features selection using the least absolute shrinkage and selection operator (LASSO) logistic regression model**


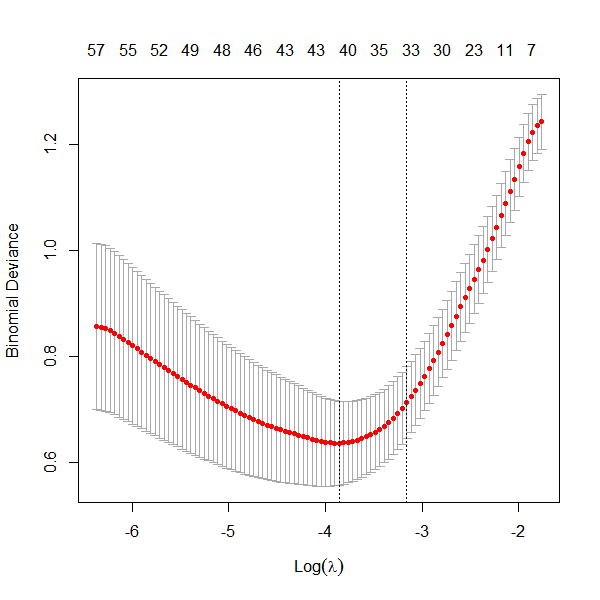

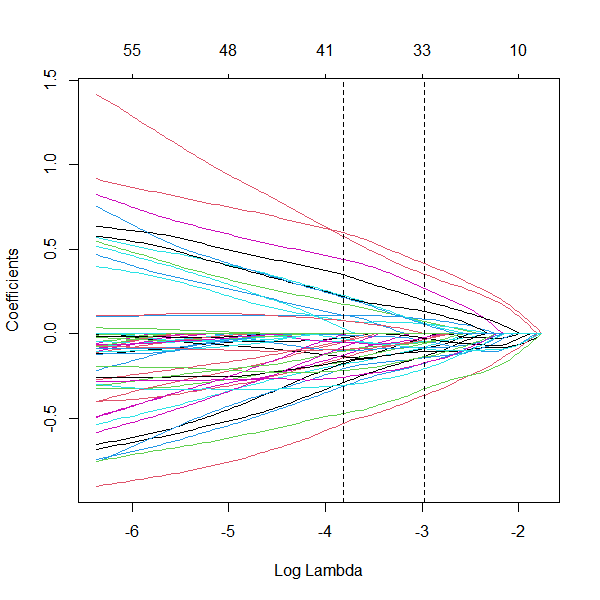


(a) (b)

**Figure S2**  DLR feature selection using the LASSO logistic regression algorithm. (a) The penalization coefficient λ in the LASSO model was tuned by the binomial deviance minimization criteria. As a result, the optimal λ of 0.0445107, was selected. (b) LASSO coefficient profiles of the 50 radiomics features. For the optimal λ, thirty-three features with nonzero coefficient were selected.
